# Evidence of Aortopathy in Mice with Haploinsufficiency of *Notch1* in *Nos3*-Null Background

**DOI:** 10.3390/jcdd2010017

**Published:** 2015-03-09

**Authors:** Sara N. Koenig, Kevin M. Bosse, Holly A. Nadorlik, Brenda Lilly, Vidu Garg

**Affiliations:** 1 The Center for Cardiovascular and Pulmonary Research and Heart Center, Nationwide Children’s Hospital, 700 Children’s Drive, Columbus, OH 43205, USA; E-Mails: Sara.Koenig@nationwidechildrens.org (S.N.K.); Kevin.Bosse@nationwidechildrens.org (K.M.B.); Holly.Nadorlik@nationwidechildrens.org (H.A.N.); Brenda.Lilly@nationwidechildrens.org (B.L.); 2 Department of Pediatrics, The Ohio State University, 700 Children’s Drive, Columbus, OH 43205, USA; 3 Department of Molecular Genetics, The Ohio State University, 484 West 12th Avenue, Columbus, OH 43210, USA

**Keywords:** ascending aortic dilation, Notch, endothelial nitric oxide, sinotubular junction effacement, smooth muscle cell lineage

## Abstract

Thoracic aortic aneurysms (TAA) are a significant cause of morbidity and mortality in humans. While the exact etiology is unknown, genetic factors play an important role. Mutations in *NOTCH1* have been linked to bicuspid aortic valve (BAV) and aortopathy in humans. The aim of this study was to determine if haploinsufficiency of *Notch1* contributes to aortopathy using *Notch1^+/−^*; *Nos3^−/−^* mice. Echocardiographic analysis of *Notch1^+/−^*; *Nos3^−/−^* mice reveals effacement of the sinotubular junction and a trend toward dilation of the aortic sinus. Furthermore, examination of the proximal aorta of *Notch1^+/−^*; *Nos3^−/−^* mice reveals elastic fiber degradation, a trend toward increased matrix metalloproteinase 2 expression, and increased smooth muscle cell apoptosis, features characteristic of aneurysmal disease. Although at a lower penetrance, we also found features consistent with aortopathic changes in *Notch1* heterozygote mice and in *Nos3*-null mice. Our findings implicate a novel role for Notch1 in aortopathy of the proximal aorta.

## 1. Introduction

Dissection and rupture of thoracic aortic aneurysms has an incidence of approximately 7 in 100,000 people per year [1]. Thoracic aortic aneurysms (TAA) are often asymptomatic, leading to the high rate of dissection and rupture. Aortic aneurysms are defined as dilation of the aorta to 50% greater than its normal. Histological examination of an aneurysmal aorta demonstrates vascular smooth muscle cell apoptosis and disorganization of the extracellular matrix (ECM) with increased collagen deposition, elastin degradation and increased levels of matrix metalloproteinases.

**Figure 1:**
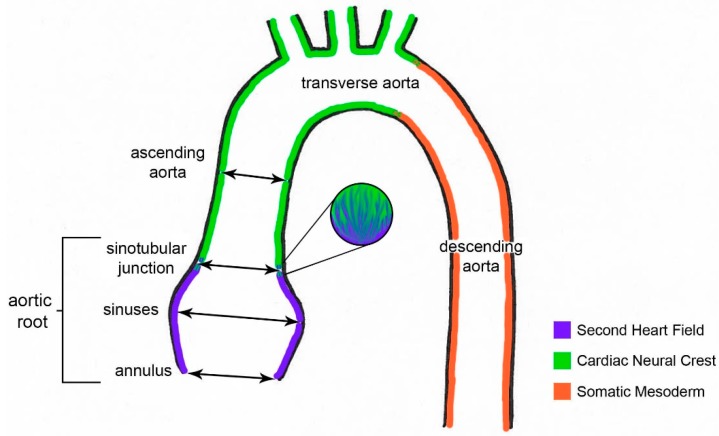
Cell lineages that contribute to the smooth muscle cells lining the ascending, transverse and descending regions of thoracic aorta are shown in schematic [31,32,33,34]. Location of echocardiographic measurements of annulus, sinus, and sinotubular junction, and ascending aorta are also shown.

Aneurysms of the thoracic aorta can occur in the following four main locations—aortic root, ascending, transverse, and descending aorta (Figure 1). Aneurysms of the aortic root can be further localized to the aortic annulus, sinuses of Valsava and the sinotubular junction (STJ) (Figure 1). The clinical phenotype of TAA in genetic syndromes suggests that aneurysms located at varying sites of the thoracic aorta may have different etiologic mechanisms [2], and this may be dictated by contributing embryonic cell lineages (Figure 1). Aneurysms involving the sinuses of Valsava and the STJ are typical for patients with Marfan syndrome [3], who harbor mutations in *FIBRILLIN1* [4,5,6,7], and in individuals with familial mutations in *ACTA2* [8] and *MYH11* [9]. While in Fabry disease (α Galactosidase A deficiency), patients present with aortic dilation of the sinuses of Valsava and/or the ascending aorta [10]. Individuals with vascular Ehlers-Danlos syndrome [11] (*COL3A1*) and Loeys-Dietz syndrome (mutations in *TGFBR1*, *TGFBR2*, and *SMAD3*) [12] present with aneurysms throughout the thoracic aorta. Patients with non-syndromic bicuspid aortic valve (BAV) [13,14] usually have aneurysms of the ascending aorta and uncommonly, will have dilation of the aortic annulus, sinuses of Valsava and STJ [15,16]. In BAV that is associated with Turner syndrome, aneurysms are consistently found in the sinuses, STJ and ascending aorta [17,18,19].

Mutations in *NOTCH1* were first reported in familial BAV and calcific aortic valve disease, and interestingly, a subset of these patients also had dilation of the ascending aorta [20], which is referred to as bicuspid aortopathy [15]. Bicuspid aortopathy is associated with histological changes similar to those observed with TAA [15]. NOTCH1 encodes a transmembrane receptor that signals through cell-cell contact and is essential for cardiovascular development [21]. Notch1 is critical for normal vascular development, as homozygote knock-out mice die at embryonic day 10.5 due to vascular endothelial defects [22,23]. Notch1 is also expressed throughout the cardiac outflow tract and has been shown to be required for proper valve development [20,24,25]. Alterations in Notch1 signaling have been reported in BAV patients with TAA [26,27,28]; however, the role of Notch1 signaling in TAAs of patients with tricuspid aortic valves is unclear [29].

We recently reported a new highly penetrant mouse model for BAV by backcrossing *Notch1^+/−^* into an endothelial nitric oxide synthase (*Nos3)*-null background [30]. The development of ascending aortic dilation has not been investigated in this murine model of BAV. Here, we show that *Notch1^+/−^*; *Nos3^−/−^* adult mice develop aortic dilation specifically with early effacement of the STJ, defined as a loss of a distinct border between the aortic sinus and proximal ascending aorta. This dilation is accompanied by histologic features of aortic aneurysms, including elastic fiber degeneration and an increase in apoptosis and a trend toward increased matrix metalloproteinase 2 (MMP2) expression in aortic smooth muscle cells. Furthermore, we find that the aortic root dilation is independent of hemodynamically significant aortic valve disease consistent with an embryologic origin for BAV-associated aortopathy. Additionally, we have found some features consistent with aortopathy in both *Nos3^−/−^* and *Notch1^+/−^* mice. *Nos3^−/−^* mice display effacement of STJ, albeit at a significantly lower frequency than *Notch1^+/−^*; *Nos3^−/−^* mice. Alternatively, histological and molecular abnormalities exist within the aorta of *Notch1^+/−^* mice, including irregular elastin fibers. These data suggest that Notch1 signaling plays a role in the development of aortopathic changes in the ascending aorta.

## 2. Experimental Section

### 2.1. Mice

All experiments were approved by the Institutional Animal Care and Use Committee at the Research Institute at Nationwide Children’s Hospital. *Nos3^−/−^* and *Notch1^+/−^* mice were genotyped as originally described [22,35]. *Nos3^−/−^* mice were bred to *Nos3^−/−^; Notch1^+/−^* mice to obtain wildtype, *Notch1^+/−^, Nos3^−/−^*, and *Notch1^+/−^*; * Nos3^−/−^* littermates.

### 2.2. Echocardiography

**Figure 2:**
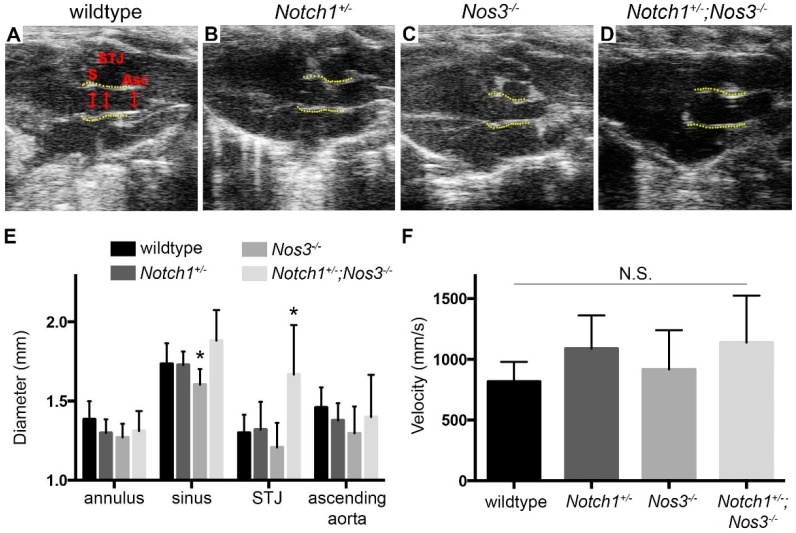
Loss of sinotubular junction in *Notch1^+/−^*; *Nos3^−/−^* mice. Representative echocardiographic images of ascending aorta at 3 months of age are shown in (**A**–**D**); S, sinus; STJ, sinotubular junction; Asc, ascending aorta. The aortic annulus, aortic sinus, STJ, and ascending aorta are outlined in yellow; (**E**) Aortic diameters at 6 months of age showing a trend of aortic sinus dilation and a significant increase in diameter of STJ in *Notch1^+/−^*; *Nos3^−/−^* mice compared to wildtype, *Notch1^+/−^,* and *Nos3^−/−^* mice. * *p*-value < 0.05 (**F**) Velocity (mean and standard deviation) across aortic valve at 6 months of age for each genotype is shown. No evidence of stenosis (defined as velocity > 2000 mm/s) was noted.

Transthoracic echocardiography was performed using a VEVO 2100 Ultrasound System as previously described [30]. Briefly, mice were sedated briefly with 3% isoflurane, and then titrated to 1%–2% isoflurane to maintain heart rate between 400 and 500 beats per minute. Aortic diameters were measured from parasternal long axis B-mode images (Figure 2) during systole at designated locations (Figure 1). Aortic flow velocity was measured with pulse-wave Doppler across the aortic valve. STJ angle was measured using the STJ as the vertex with arms extending to the sinus and ascending aorta. The greatest angle of the left or right side was chosen. All data points are an average of three measurements.

### 2.3. Tissue Fixation and Histology

Experimental animals were euthanized and mouse hearts were perfused in a consistent manner with 1 mL of 10% formalin using a 5 mL syringe into the left ventricle*.* Serial sections were stained with hematoxylin and eosin, elastin stain kit (Sigma HT25A), Masson’s trichrome, and immunohistochemistry (Santa Cruz ImmunoCruz sc-2018 or Cell Signaling 8114, 8125, 8112, and 8059) with primary antibodies against MMP2 (Abcam, ab37150, 1:500), Caspase-3 (Cell Signaling, 9661, 1:300), activated Notch1 (abcam, ab8925, 1:500), and α-Smooth Muscle Actin (Sigma Aldrich, A2547, 1:500). Integrated density was determined using the IHC Toolbox plugin for ImageJ [36].

### 2.4. Elastin Quantification and Basic Morphometrics

Images of elastin stained tissue sections were measured (3 images per animal), and measurements were repeated 5 times in different areas of each image. (*n* = 3 wildtype, 3 *Notch1^+/−^,* 3 *Nos3^−/−^,* and 4 *Notch1^+/−^*; *Nos3^−/−^* mice). Gross images of the aorta were measured at the aortic root, ascending aorta, aortic arch, and descending aorta (*n* = 4 per genotype). All measurements were made in a blinded fashion in regards to the genotype.

### 2.5. TGF-β1 ELISA

Blood was collected from the left ventricle prior to perfusion fixation at the time of euthanasia, and platelet-poor plasma was isolated from the blood. The ELISA kit was purchased from R&D systems (MB100B). TGF-β1 was activated in plasma (according to instructions) prior to assay.

### 2.6. Statistics

Statistical analysis was performed using Student’s t-tests and one-way ANOVA for echocardiographic and gross diameter measurements and basic morphometrics, and Fisher’s exact test for incidence of STJ effacement. Statistical significance was determined based on a *p*-value < 0.05.

## 3. Results and Discussion

### 3.1. Effacement of Sinotubular Junction and Aortic Root Dilation in Notch1^+/−^; Nos3^−/−^ Mice

In order to determine if *Notch1^+/−^*; *Nos3^−/−^* mice develop aneurysms of the ascending aorta, we bred *Nos3^−/−^* mice to *Nos3^−/−^*; *Notch1^+/−^* compound heterozygotes to obtain wildtype, *Notch1^+/−^*, *Nos3^−/−^* and *Notch1^+/−^*; *Nos3^−/−^* littermates. As expected, Notch1 expression was decreased in *Notch1^+/−^* and *Notch1^+/−^*; *Nos3^−/−^* mice by immunohistochemistry (Supplemental Figure 1). Echocardiographic measurement of the ascending aorta of *Notch1^+/−^*; *Nos3^−/−^* mice at 3 months of age revealed effacement of the STJ in 5 out 5 animals (100%) (Table 1, Figure 2D). Interestingly, effacement of the STJ was also seen in a subset of *Notch1^+/−^* and *Nos3^−/−^* mice but at a statistically significant lower frequency (Table 1, Figure 2). At this time point, there was no difference in mean aortic diameter between genotypes [37]. Follow up echocardiographic examination at 6 months of age demonstrated a similar frequency of STJ effacement (Table 1, Supplementary Figure 2) except for one additional *Nos3^−/−^* mouse had developed STJ effacement. Consistent with this, the aortic sinus-ascending aortic angle was increased in *Notch1^+/−^*; *Nos3^−/−^* mice at 6 months of age (Supplementary Figure 2Q). At 6 months, the diameter of the STJ was significantly higher in *Notch1^+/−^*; *Nos3^−/−^* mice than controls (Figure 2E). The aortic sinus significantly larger in the *Notch1^+/−^*; *Nos3^−/−^* mice than the *Nos3^−/−^* mice (*p* value = 0.0016), but this trend was not statistically significant in wildtype or *Notch1^+/−^* mice (*p* value > 0.1). The velocity across the aortic valve was not statistically different among all four genotypes (Figure 2F).

**Table 1:** Effacement of Sinotubular Junction by Genotype. Fraction of mice with STJ effacement is shown at 3 and 6 months of age, * *p* value <0.05.

	Wildtype	*Notch1^+/−^*	*Nos3^−/−^*	*Notch1^+/−^*; *Nos3^−/−^*
3 months	0/8 (0%)	1/8 (12.5%)	3/11 (27.3%)	5/5 (100%) *
6 months	0/8 (0%)	1/8 (12.5%)	4/11 (36.4%)	5/5 (100%) *

### 3.2. Notch1^+/−^; Nos3^−/−^ Mice Display Gross and Histologic Evidence of Aortopathy

**Figure 3:**
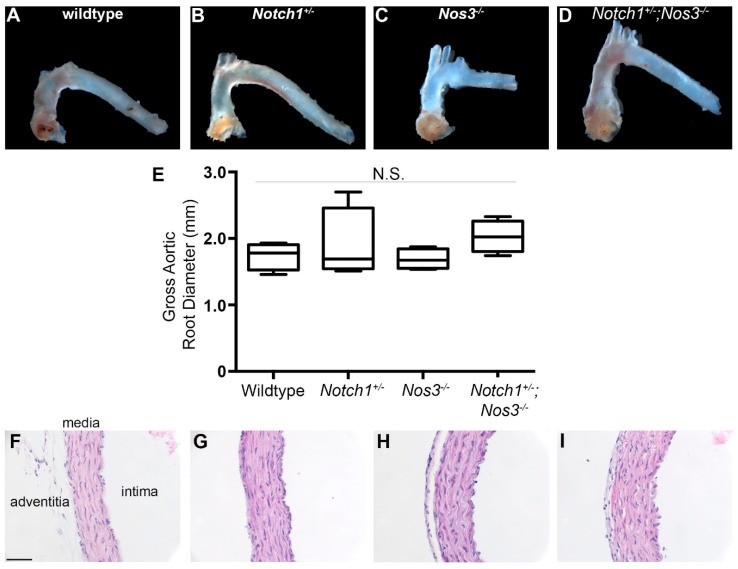

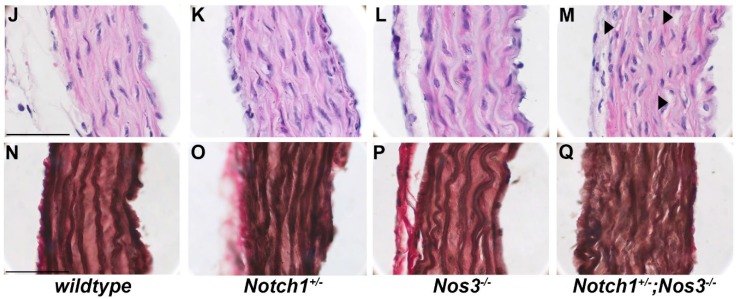
Dilation of proximal ascending aorta in *Notch1^+/−^*; *Nos3^−/−^* mice. Representative gross images of thoracic aorta at 8 months are shown in (**A**–**D**); (**E**) Quantification of gross aortic root diameter showing a trend for dilation in *Notch1^+/−^*; *Nos3^−/−^* mice (*n* = 4 per genotype); N.S., not significant; (**F**–**M**) Hematoxylin and eosin stained aortic sections at 400x and 1000x, respectively (**F**–**I**, **J**–**M**), showing areas of presumed SMC loss in *Notch1^+/−^*; *Nos3^−/−^* (**M**, arrowheads). (**N**–**Q**) Representative images of elastin stained aortic cross sections at 1000x, illustrating degraded elastin in the *Notch1^+/−^* and *Notch1^+/−^*; *Nos3^−/−^* mice. Scale bar equals 50 microns.

Gross examination of the proximal ascending aorta at 8 months of age demonstrated a trend toward dilation of the aortic root (mean diameter = 2.03 ± 0.121 mm) in *Notch1^+/−^*; *Nos3^−/−^* mice (Figure 3A–E), but this was not statistically significant when compared to littermate controls. Measurements did reveal one out of four *Notch1^+/−^* mice with a grossly dilated aortic root (2.7 mm in diameter). Histologic examination of the aorta in all four genotypes was performed (Figure 3F–M). It demonstrated aortic wall abnormalities reminiscent of medial degeneration (as reviewed in [38]) in *Notch1^+/−^*; *Nos3^−/−^* mice, indicated by increased “empty” space between cells presumably where smooth muscle cell loss has occurred (Figure 3M, arrowheads). This phenotype was not observed in wildtype, *Nos3^−/−^*, and *Notch1^+/−^* littermates. In addition, we observed diffuse elastin staining in the aorta of *Notch1^+/−^* mice similar to the *Notch1^+/−^*; *Nos3^−/−^* murine aortas. Additionally, *Notch1^+/−^*; *Nos3^−/−^* mice had elastic fiber degradation, identified by an overall disorganization of the layers (Figure 3Q). The elastin fibers were normal in *Nos3^−/−^* and wildtype littermates (Figure 3N–Q).

**Figure 4:**
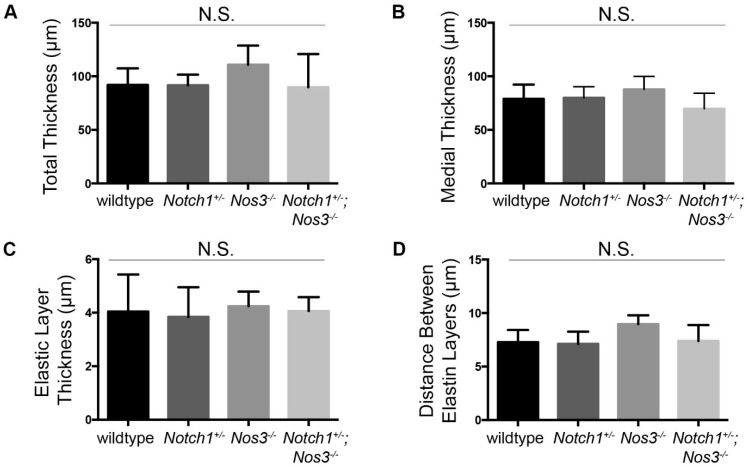

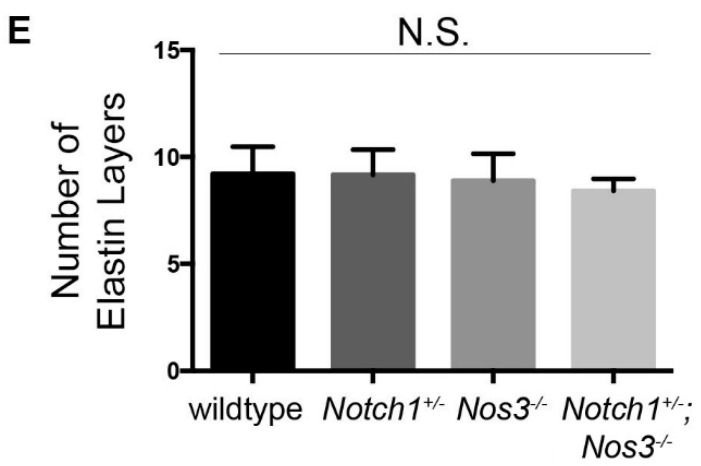
No difference in basic morphometrics of the aortic wall in *Notch1^+/−^*; *Nos3^−/−^* mice as compared to littermate controls. Quantification of total aortic wall thickness (**A**); medial thickness (**B**); elastic layer thickness (**C**); distance between elastic layers (**D**); and number of elastin layers (**E**). N.S., not statistically significant. N = 3 per for each genotype.

Although evidence of elastin disorganization was observed, there were no quantifiable differences between genotypes in total aorta thickness, medial thickness, elastic layer thickness, distance between elastic layers, and number of elastic layers (Figure 4).

### 3.3. Notch1^+/−^; Nos3^−/−^ Aortas Display Molecular Evidence of Aortopathy

**Figure 5:**
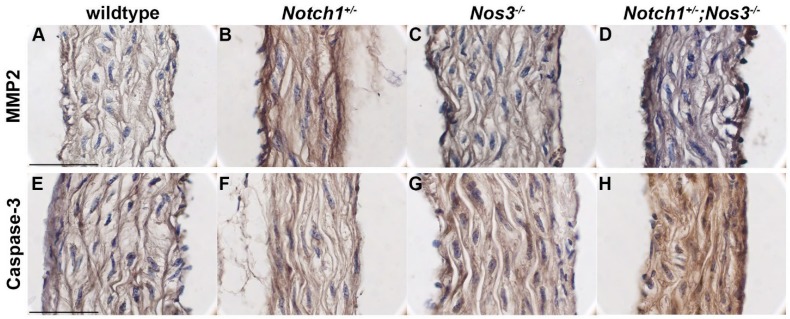

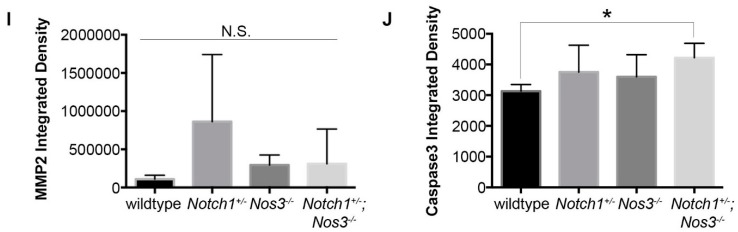
Immunohistochemical evidence of aortopathy in *Notch1^+/−^*; *Nos3^−/−^* mice. MMP2 protein expression is increased in *Notch1^+/−^*; *Nos3^−/−^*, *Nos3^−/−^*, and *Notch1^+/−^* in comparison to wildtype (**A**–**D**, **I**). Caspase-3 protein expression is increased in *Notch1^+/−^*; *Nos3^−/−^* in comparison to littermates, * *p* value < 0.05 (**E**–**H**, **J**). Scale bar equals 50 microns.

To determine if the dilated aorta in *Notch1^+/−^*; *Nos3^−/−^* mice displayed evidence of ECM degradation, we examined the expression of MMP2, an MMP (matrix metalloproteinase) that is commonly overexpressed in aneurysms [39]. In the aorta, MMP2 expression was found to be increased in *Notch1^+/−^*; *Nos3^−/−^*, and the *Notch1^+/−^*; *Nos3^−/−^* aortas in comparison to wildtype littermates although it did not reach statistical significance (Figure 5A–D). Another characteristic of aortopathy is smooth muscle cell apoptosis, and therefore, we examined the levels of caspase-3. In the ascending aorta of *Notch1^+/−^*; *Nos3^−/−^* mice, we found increased expression levels of caspase-3 in comparison to littermates (Figure 5E–H).

### 3.4. Significance

Thoracic aortic aneurysms are a significant cause of morbidity and mortality in adults, and are particularly prevalent in the BAV population. There is substantial interest in discerning the hemodynamic and genetic influences in aortic aneurysm development in these patients. In this study, we have identified a specific mouse model (*Notch1^+/−^*; *Nos3^−/−^*) which develops dilation of the aortic sinus and effacement of the STJ, characteristics which are proposed to have genetic origins in humans [15]. STJ effacement is usually not an isolated clinical phenomenon, and it is possible that significant aortic dilation would be observed if these mice were further aged. We have also, for the first time, described pathologic changes suggestive of an ascending aortopathy in a subset of *Notch1* heterozygote and *Nos3*-null mice, with effacement of the STJ and a trend toward increased MMP2 expression, although no evidence of dilation was observed in either *Notch1^+/−^* mice and *Nos3^−/−^* at 6 months of age. Overall, these findings suggest inherent defects in the aorta of *Notch1^+/−^* and *Nos3^−/−^* mice which when combined result in dilation of the aortic root, characterized by classic signs of aneurysm such as medial and elastic fiber degradation and an increased expression of MMP2 and caspase-3.

Effacement of the STJ is noteworthy when considering the embryonic smooth muscle cell lineages that contribute to the thoracic aorta. SMCs in the aortic root are primarily derived from the anterior heart field [32], whereas SMCs in the ascending aorta are primarily derived from the cardiac neural crest [33] (Figure 1). It has been suggested by several investigators that SMCs have different signaling mechanisms that are dependent on their embryonic lineage [31]. While the derivation of the SMCs in the thoracic aorta is split, the endothelial cells are all derived from mesoderm-derived angioblasts (reviewed in [40]). The endothelial lining of the aorta is critical to the maintenance of SMCs and the overall aortic wall. Endothelial cells provide extracellular matrix, inhibit SMC proliferation, and release nitric oxide to aid in vasodilation. Notch1 is predominantly located in endothelial cells throughout the adult cardiovascular system [41]. It is possible that the loss of endothelial nitric oxide and Notch1 specifically affects the STJ because at this point of lineage divergence, SMC response to endothelial cell signals differs. For example, blood flow patterns normally differ between the aortic root and the ascending aorta [42], and endothelial cells signal to SMCs in response to flow patterns [43]. Because the STJ is a heterogeneous population of anterior heart field-derived and cardiac neural-crest derived SMCs (Figure 1) and endothelial cell-SMC signaling is lineage specific, the aortic wall at the STJ could be more susceptible to disrupted endothelial cell signaling than the aortic root or the ascending aorta, both of which have homogenous SMC populations.

TGF-β expression is known to be increased in many TAA patients [44], although its exact mechanism of action in aneurysmal development is unclear [45]. Total (active and inactive) circulating TGF-β1, a transcriptional target of TGF-β signaling, was assayed in the *Notch1^+/−^*; *Nos3^−/−^* mice, but no clear differences were found (Supplemental Figure 3A). Although MMP2 expression, a transcriptional target of the TGF-β signaling pathway, was increased in our mouse model, we did not find changes in the expression of phosphorylated SMAD2 (Supplemental Figure 3B). It is possible that with age and more severe disease, an increase in circulating TGF-β1 would be more readily measureable.

## 4. Limitations

While our study is the first to describe an aortopathic phenotype in *Notch1^+/−^*; *Nos3^−/−^* mice, it has several limitations. First, the mice were only aged to eight months and therefore the identified differences in the compound mutant mice were not dramatic. Additional studies examining the aorta after pharmacologic treatment to induce hypertension (angiotensin II or phenylephrine infusion) of *Notch1^+/−^* and *Notch1^+/−^*; *Nos3^−/−^* may give a more definitive picture of the role on Notch1 in this disease. Secondly, it has been suggested that the more common location of aneurysms in BAV patients which are localized to the ascending aorta are due to hemodynamic effects, while those localized to the aortic root are attributed to genetic factors (Figure 1, reviewed in [15]). Consistent with this notion, the *Notch1^+/−^*; *Nos3^−/−^* mice did not demonstrate abnormal hemodynamics (Figure 2F). We were unable to examine the aortic valve morphology in the *Notch1^+/−^*, *Nos3^−/−^* and *Notch1^+/−^*; *Nos3^−/−^* mice, a significant limitation of this study. In our experience, the most reliable method to characterize aortic valve morphology is to dissect the aortic valve with an attached cuff of the proximal aorta as this allows for direct visual inspection and mechanical manipulation to determine cusp fusion [30]. This type of analysis does not allow for histological examination of the diseased portion of the aorta since it was directly adjacent to the valve. Lastly, our model involves the disruption of the Notch1 signaling pathway and therefore these findings may not be relevant to all BAV associated aortopathy.

## 5. Conclusions

In conclusion, this study describes a new mouse model of aortopathy that includes effacement and dilation of the STJ and a trend toward dilation of the aortic sinus, although this was not significant. Our murine model suggests that STJ effacement may be an early indicator of aortopathy that has embryologic origins and molecular basis. This model has histological and molecular features consistent with ascending aortic aneurysms, including elastin degradation, smooth muscle cell apoptosis, and increased MMP2 expression. Additionally, we have described more subtle and less penetrant pathological changes in the aorta of the *Nos3^−/−^* and *Notch1^+/−^* mice.

## Supplementary Materials

Supplementary materials can be accessed at: http://www.mdpi.com/2308-3425/2/1/0017/s1.
