# Supplementary material for: Evidence of Aortopathy in Mice with Haploinsufficiency of Notch1 in Nos3-Null Background
Source: J Cardiovasc Dev Dis. 2015 Mar 9;2(1):17–30. doi: 10.3390/jcdd2010017 (PMC4407710; doi:10.3390/jcdd2010017)
Supplement: Supplementary file 1 [file jcdd-02-00017-s001.pdf]

Supplementary Materials

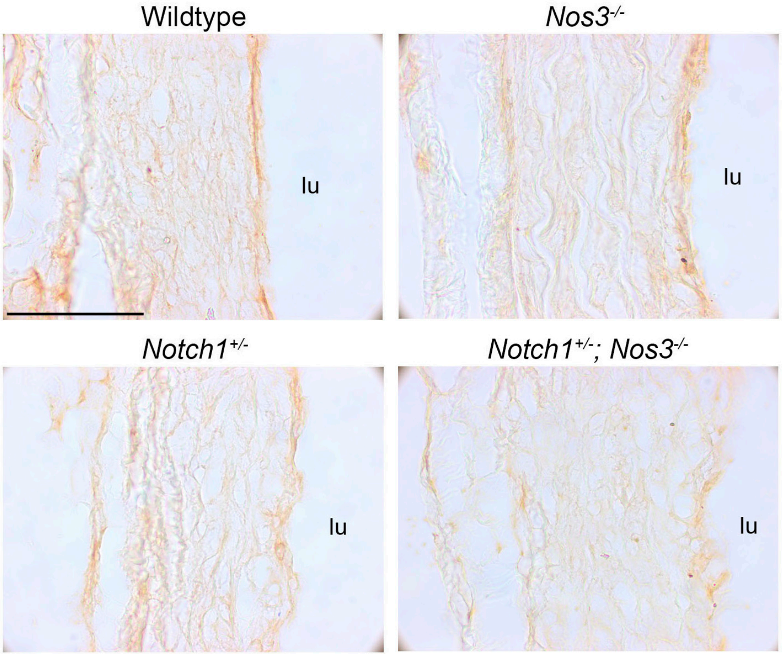

Figure S1. Expression of Notch1 intracellular domain (activated Notch1) by immunohistochemistry is decreased in endothelial cells lining the lumen (lu) of *Notch1*$^{+/-}$and *Notch1*$^{+/-}$; *Nos3*$^{-/-}$ mice as compared to wildtype and *Nos3*$^{-/-}$. Scale bar = 50 microns.

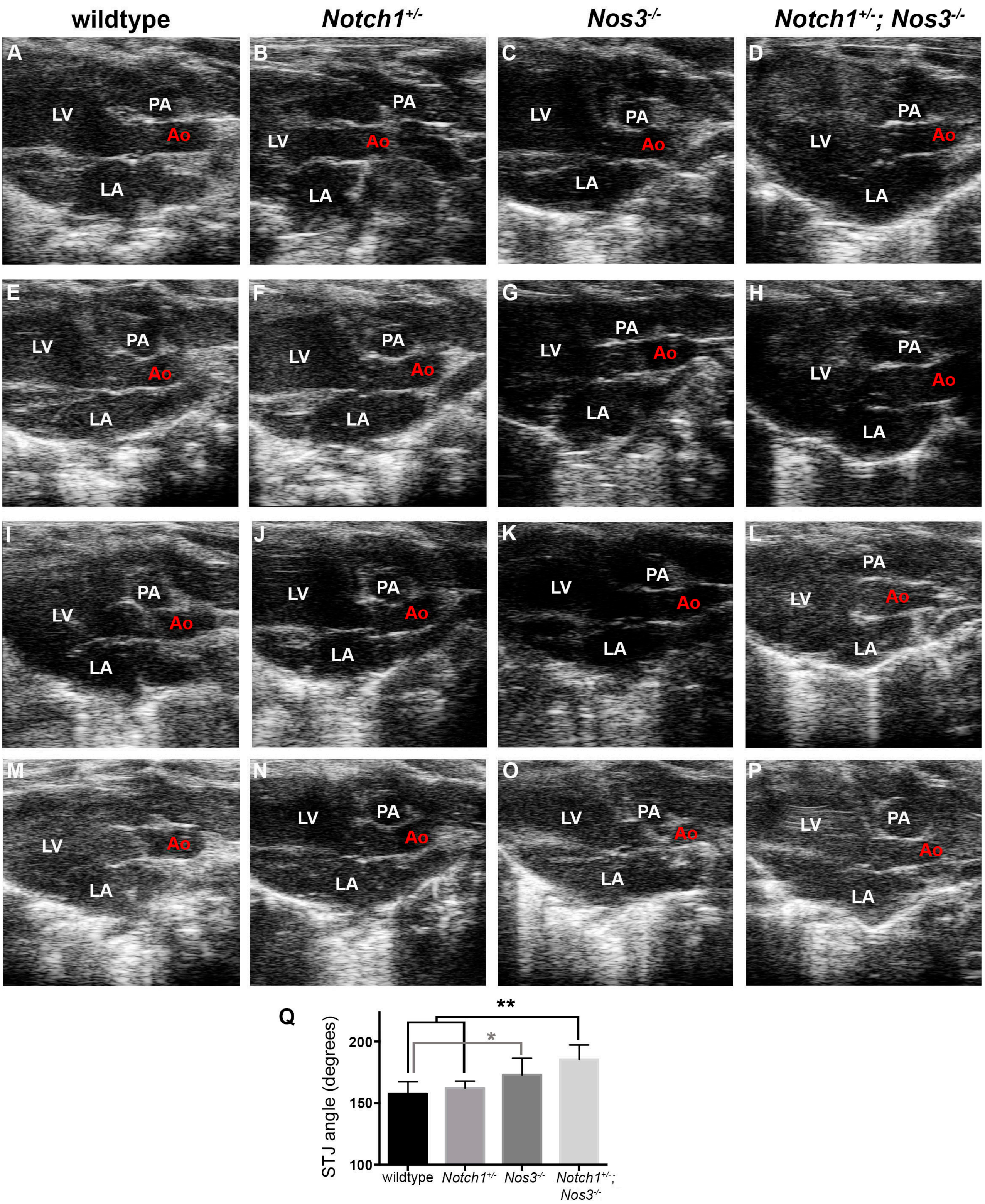

Figure S2. Representative echocardiographic images of ascending aorta from 6 month old mice of each genotype (**A**–**P**). Each image represents a different animal (4 mice per genotype are shown). LV, left ventricle; Ao, aorta. (**Q**) Quantified aortic sinus-ascending aorta angle in 6 month old mice (* p value < 0.05, ** p value < 0.01).

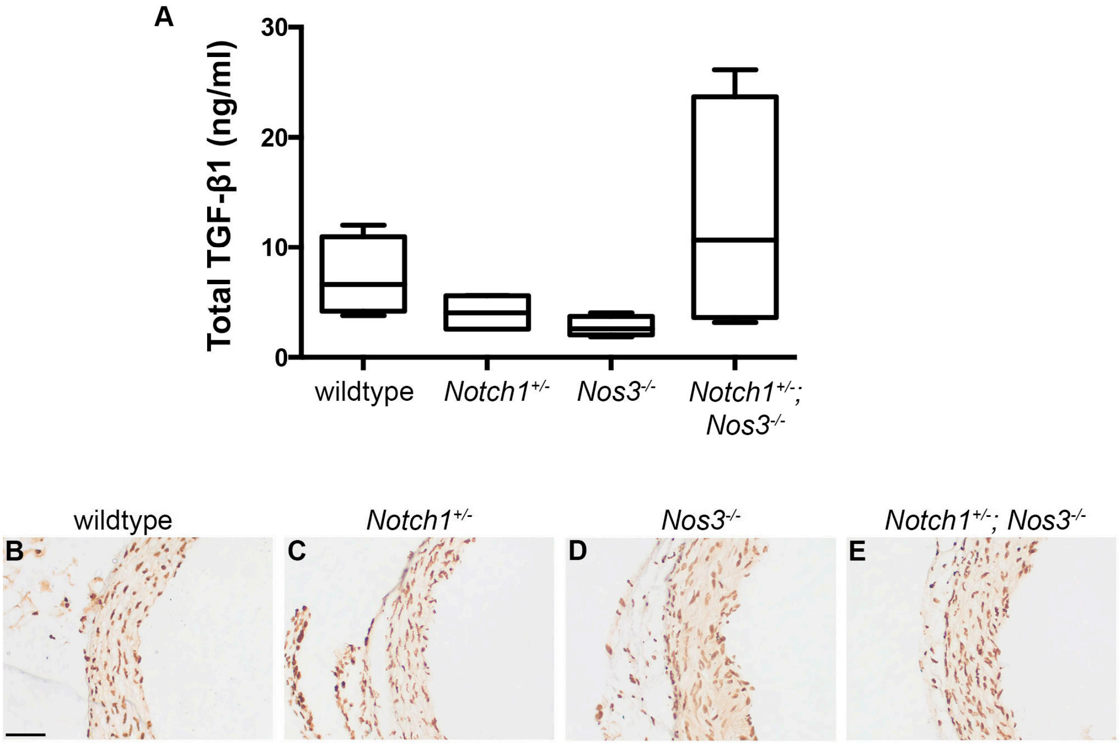

Figure S3. TGF-β signaling is not consistently elevated in *Notch1*$^{+/-}$; *Nos3*$^{-/-}$ mice. (**A**) Serum levels of total circulating TGF-β1 in serum from 8 month old mice of all four genotypes demonstrate no statistically significant differences. (**B–E**) Expression of phosphorylated SMAD2 by immunohistochemistry in the aortic wall is unchanged between genotypes. Aortas of 8 month old mice were examine at *n* = 3 per genotype.
